# Type 4 Tibial Tuberosity Avulsion Fractures: Surgical Treatment Early Outcomes and a Presentation of the Distal Cortical Fixation

**DOI:** 10.3390/jcm13195695

**Published:** 2024-09-25

**Authors:** David Segal, Michael Dillenkofer, Eric J. Wall, Junichi Tamai

**Affiliations:** 1 Division of Orthopaedic Surgery, Cincinnati Children’s Hospital Medical Center, 3333 Burnett Ave, MLC 2017, Cincinnati, OH 45229, USA; 2 Division of Orthopedic Surgery, WellSpan Health York Hospital, 1001 S. George St., York, PA 1740, USA

**Keywords:** Ogden, Watson–Jones, technique, Salter–Harris

## Abstract

**Background**: The most published surgical technique for fixating Type 4 (Salter–Harris II) tibial tubercle avulsion fractures is uni-cortical in nature, and stability is suboptimal. This study presents a technique modification that is consistent with AO principles, by which the screws are aimed distally to purchase the posterior cortex of the distal fragment. This technique is defined as a “Distal Cortical Fixation”. This modification has not been studied to date and harbors potential advantages. We aimed to assess the safety and efficacy of surgical fixation techniques for the above-mentioned fractures and to describe the new modification. **Methods**: A retrospective review was conducted at a level 1 children’s hospital for surgically treated Salter–Harris II tibial tubercle fractures. Inclusion criteria were patients who sustained Salter–Harris II tibial tubercle avulsion fractures and were documented to reach one of two radiographic endpoints: union (regardless of alignment) or non-union that necessitated additional interventions. Medical records and radiographic studies were analyzed for fracture union and alignment. A comparative analysis was conducted to evaluate outcomes based on different fixation techniques that included Distal Cortical Fixation, a Proximal Screw Technique, and a crossed or multiple screws/pins construct. **Results**: A total of 37 patients were included with a mean age of 14.8 ± 1.2 years, with 34/37 (91.9%) being male. The most common procedure was a 1 to 3 screw fixation with a Distal Cortical Fixation (*n* = 21 (56.75%)), followed by a Proximal Screw Technique (*n* = 8, 21.62%), and a crossed or multiple screws/pins construct (*n* = 8, 21.62%). There was no difference between the groups in medical history and demographic features. The mean follow-up duration was 35.17 ± 36.79 weeks. There were no non-unions, and only a minimal change in the sagittal and coronal alignment (0.4 ± 1.94 (*p* = 0.872) and 0.53 ± 3.51 (*p* = 0.296) degrees, respectively) was noted and was not associated with the surgical technique. **Conclusions**: The surgical treatment of Salter–Harris II tibial tubercle avulsion fractures, including Distal Cortical Fixation, was presented and was found to provide satisfactory union rates on a short term follow up.

## 1. Introduction

**Figure 1:**
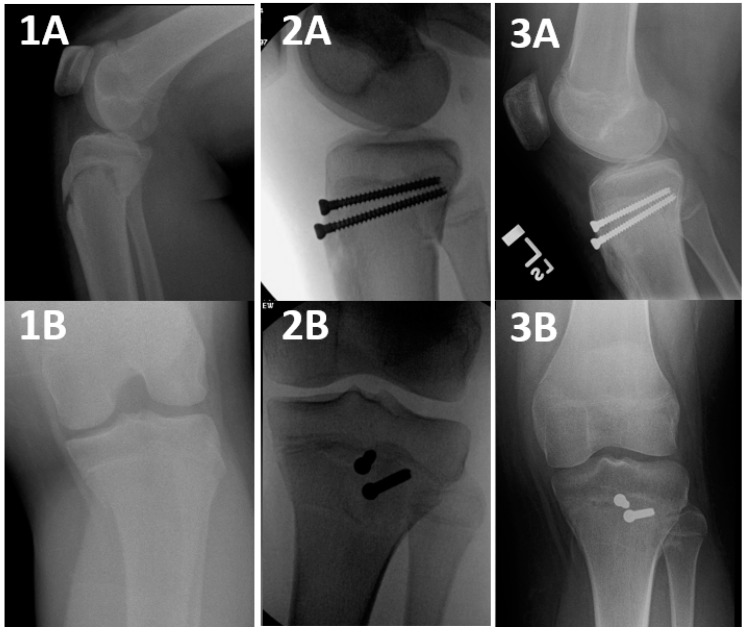
A Salter–Harris II tibial tuberosity avulsion fracture, treated surgically with a Proximal Screw Technique (PST). Salter–Harris II TTAF stands for a modified Watson–Jones type 4 TTAF fracture that propagates through the entire proximal tibial physis, starting at the anterior cortex and progressing all the way to the posterior cortex, with a posterior Thurston–Holland component of various sizes. A PST is a fixation that captures the proximal fragment at its anterior cortex, with or without the posterior cortex fixation, and captures the distal fragment at its cancellous bone only. **1A** and **1B**: Preoperative, **2A** and **2B**: intraoperative: **3A** and **3B**: postoperative X ray imaging studies.

Tibial tuberosity avulsion fractures (TTAF) are unique for the pediatric and adolescent populations [1,2,3,4]. These fractures are the consequence of the patellar tendon pullout force in a state where the proximal tibia physis is still active, leading to a failure at this weak spot [5]. These fractures were primarily classified by Watson–Jones into three types [5] and have been further divided by Ogden et al. [2], Ryu et al. [6], and McKoy et al. [1] into five distinct categories (modified Watson–Jones classification (mWJ)) [4]. Type 4 mWJ (Figure 1) is a Salter–Harris (SH) II TTAF that propagates through the entire proximal tibial physis, starting at the anterior cortex and progressing all the way to the posterior cortex, with a posterior Thurston–Holland component of various sizes [6]. The latter subtype, although considered rare, has been constantly increasing in incidence [7,8,9].

The goal of surgical fixation of SH II TTAF is restoring alignment and stabilizing the fracture fragments [1,3,7,8,10,11,12,13,14]. Various fixation techniques have been described, including the use of screws, plates, or K wires [1,6,9,10,11,12,13,14]. However, there remains a paucity of studies that compare fixation techniques for this distinct subgroup of fractures. The most frequently published surgical fixation technique thus far for these fractures has been a screws construct, where the screws were inserted through the anterior cortex of the proximal fragment, traversed through the distal fragment’s metaphyseal cancellous bone and incorporated into the posterior cortex of the proximal fragment (i.e., Proximal Screw Technique: PST, Figure 1). This provided a cortical fixation of the proximal fragment while capturing the distal fragment only at the weak, hollow, cancellous bone [3,9,10,11,12,13].

**Figure 2:**
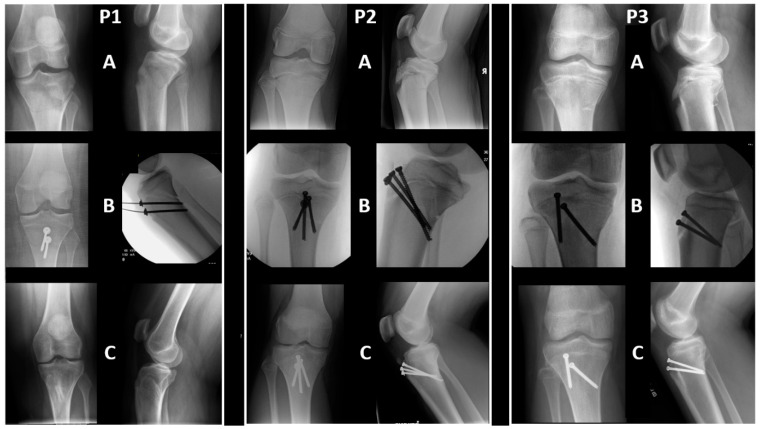
Salter–Harris II tibial tuberosity avulsion fractures (TTAF), treated surgically with a Distal Cortical Fixation (DCF). Salter–Harris II TTAF stands for a modified Watson–Jones type 4 TTAF fracture that propagates through the entire proximal tibial physis, starting at the anterior cortex and progressing all the way to the posterior cortex, with a posterior Thurston–Holland component of various sizes. A DCF is a fixation that captures the proximal fragment at its anterior cortex and captures the distal fragment at its posterior cortex. P1, P2, and P3 were three different patients. (**A**–**C**): Pre, intra (or immediately after), and final follow-up X-ray images, respectively.

**Figure 3:**
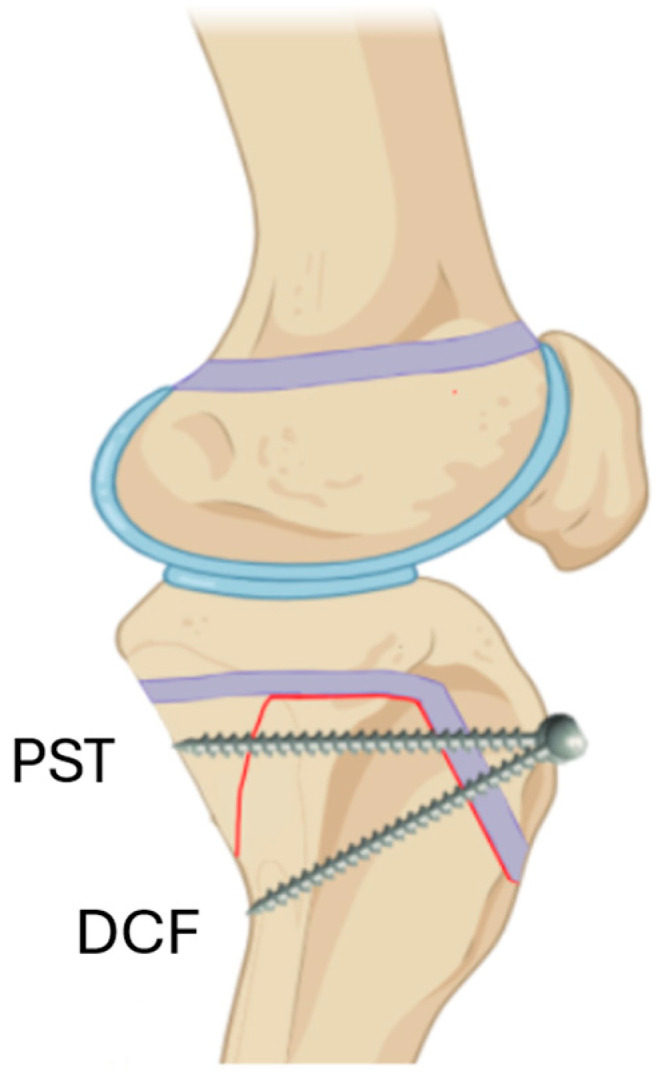
A graphical representation of a Proximal Screw Technique (PST) screw and a Distal Cortical Fixation (DCF) screw for treating a Salter–Harris II tibial tuberosity avulsion fracture. The PST screw captures two cortices of the proximal fragment and none of the distal fragment and is, therefore, a “bi-cortical screw”. The DCF screw captures each fragment at its cortex and is, therefore, a “bi-cortical fracture fixation”. Adapted from “Knee (lateral, bent, silhouette)”, by BioRender.com (2024). Retrieved from http://app.biorender.com/biorender-templates, accessed on 15 April 2024.

In the authors’ institute, these fractures have often been fixated with the screws similarly starting at the anterior cortex of the proximal fragment but directed distally (instead of parallel to the joint line), aiming to purchase the distal fragment at its posterior cortex, and thus capturing each fragment at their cortices (i.e., Distal Cortical Fixation: DCF, Figure 2 and Figure 3). The goal of using DCF was to purchase the tibial shaft into a strong, cortical bone instead of at a weak, cancellous bone. This technique, as opposed to PST, is more adequately aligned with the Association of Osteosynthesis (AO) principles for inter-fragmentary fracture fixation with screws [15], which purchase both fragments on their cortices.

Although allegedly a minor modification, it represented a fundamentally different understanding of how the forces that act on these fractures would be most effectively countered. We aimed to assess the safety and efficacy of surgical fixation techniques for the above-mentioned fractures and to describe a new technique modification (DCF) that is consistent with AO principles. Our hypothesis was that the DCF was not inferior in safety and efficacy when compared to the other fixation techniques.

## 2. Materials and Methods

This retrospective study was approved by the institutional ethical review board. A waiver of informed consent was granted. The authors utilized a computerized screening to identify patients who sustained an SH II TTAF with subsequent surgical fixation at a single level 1 children’s hospital between 1 November 2010 and 1 November 2023 under the Current Procedural Terminology (CPT) code 27540 [16]. This code has been historically used for the surgical treatment of all TTAF in this institution. Imaging studies (X-ray (XR), computed tomography (CT), and magnetic resonance imaging (MRI)) were manually reviewed to classify the fractures according to the mWJ (Ogden) classification [8]. Inclusion criteria were children who sustained an SH II TTAF and were documented to reach one of two radiographic endpoints: union (regardless of alignment) or non-union that necessitated additional interventions. Union was defined as a continuation of the cortical bone over the fracture site over at least 3 planes on follow-up XR imaging studies. Exclusion criteria were patients who did not undergo surgical fixation or fractures that extended through the articular surface [10].

The electronic medical records were reviewed to collect demographic and medical variables (Table 1). XR and fluoroscopy imaging studies were reviewed to evaluate the fracture type and pattern, alignment, fusion, implant type, position and trajectory, and cortical purchase on the bone distal to the fracture. Measurements were standardized and performed by a pediatric orthopedic surgery fellow and a fellowship-trained pediatric orthopedic surgeon.

To categorize surgical techniques, a few methods were defined: 1. DCF, 2. PST, 3. Crossed pins/screws in which surgeons used divergent lateral to medial (and visa-versa) K wires or screws’ construct. 4. “Multiple” where multiple screws were inserted in various orientations to obtain multi-directional stability. When at least one screw captured two cortices (either on the same fragment or on both fragments), the construct was regarded as bi-cortical. This definition has been used in similar previous studies [1,6,9,10,11,12,13,14] and was utilized in the current study for consistency purposes. The surgical technique in each case was chosen at the surgeon’s discretion. Implant removal was considered only once union was appreciated in the follow-up XR imaging studies. Pins were normally removed 6–8 weeks postoperatively. Screws were removed at a later stage, if at all, when hardware prominence-related symptoms occurred at the discretion of the surgeons and the patients. Analyzing the rate of implant irritation and removal was beyond the scope of this study since it required a follow-up duration that was longer than defined for the current investigation. For the same reason, the authors were limited in analyzing the final range of motion, clinical scores, and adverse effects that are related to growth disturbances.

### Statistical Analysis

Standard descriptive statistics were used to characterize the patient sample and radiographic features. Continuous variables are presented with means and standard deviations. Categorical variables are presented with numbers and rates. Comparisons of categorical variables between subgroups were completed using the Fisher exact test. Comparisons of continuous variables were completed using the Mann–Whitney U test and the Kruskal–Wallis test. A multivariable regression analysis was planned to identify independent predictors for the outcome measure but was not executed considering similar outcomes in the various surgical techniques. An SPSS 29.0 software (Armonk, NY, USA, IBM Co.) was used for the statistical analysis. A *p*-value of 0.05 and a power of 0.8 were defined for significance.

## 3. Results

**Figure 4:**
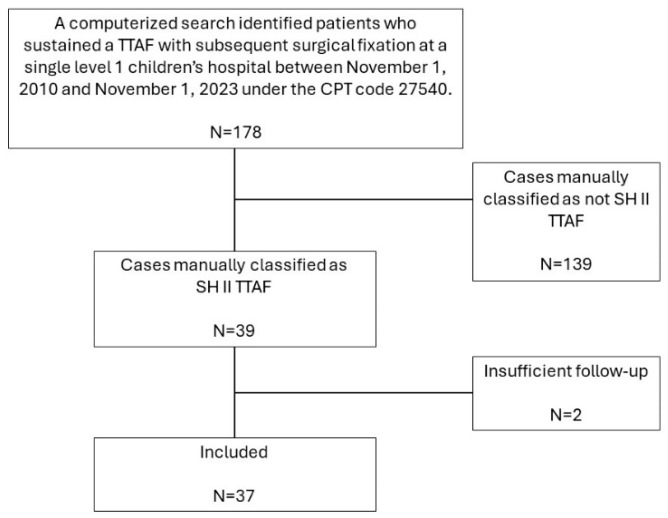
Patient inclusion flow chart. TTAF: Tibial tuberosity avulsion fracture. CPT: Current procedural terminology. SH: Salter–Harris.

A total of 178 cases were identified by the computerized search, of which 39 cases were manually classified as SH II TTAF. Of those, one patient was lost to follow-up before reaching the defined study endpoints, and one patient underwent surgical fixation just a few days before the study data were obtained and, therefore, did not have sufficient follow-up. Eventually, 37 patients (37 fractures) with a mean age of 14.88 ± 1.35 years, 18 (85.7%) males, were included in the study (Figure 4, Table 1 and Table 2).

**Figure 5:**
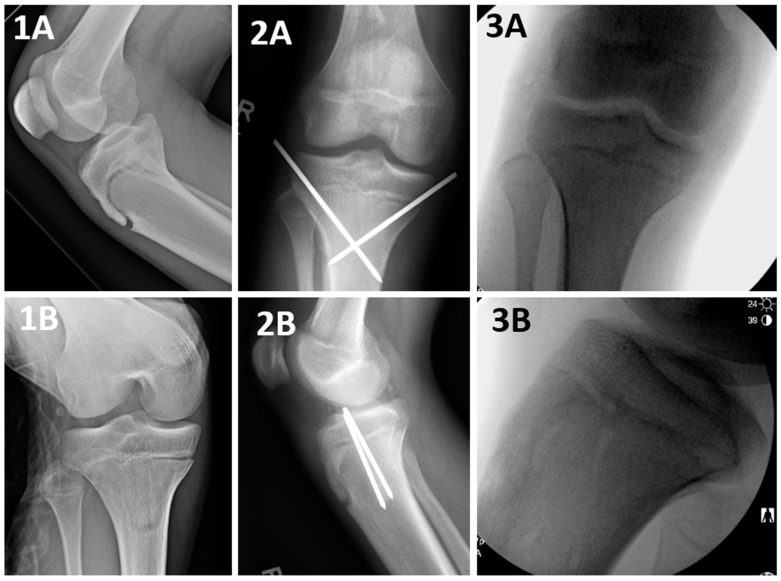
A Salter–Harris II tibial tuberosity avulsion fracture, treated surgically with a crossed pins construct. Salter–Harris II TTAF stands for a modified Watson–Jones type 4 TTAF fracture that propagates through the entire proximal tibial physis, starting at the anterior cortex and progressing all the way to the posterior cortex, with a posterior Thurston–Holland component of various sizes. A crossed pins construct is a fixation that captures the proximal fragment at its medial and lateral cortices and the distal fragment at its medial and lateral cortices. **1A** and **1B**: Preoperative, **2A** and **2B**: postoperative, **3A** and **3B**: following hardware removal X ray imaging studies..

**Figure 6:**
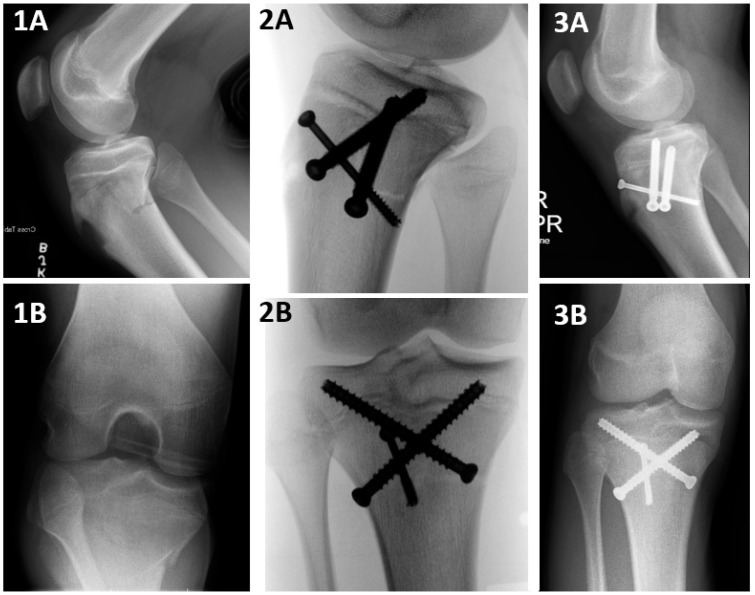
A Salter–Harris II tibial tuberosity avulsion fracture, treated surgically with a multiple-direction screw construct. Salter–Harris II TTAF stands for a modified Watson–Jones type 4 TTAF fracture that propagates through the entire proximal tibial physis, starting at the anterior cortex and progressing all the way to the posterior cortex, with a posterior Thurston–Holland component of various sizes. A multiple-direction screw construct is a fixation that captures the proximal and the distal fragment at their cortices in various locations. **1A** and **1B**: Preoperative, **2A** and **2B**: intraoperative, **3A** and **3B**: postoperative X ray imaging studies.

The main surgical technique was a DCF (Figure 2), followed by a PST (Figure 1), both utilizing 1–3 screws. An additional group of patients (*n* = 8, 21.62%) was treated with a crossed screws/pins construct (Figure 5) or multiple screws (Figure 6), which were grouped for analysis purposes due to their low number count (Table 1 and Table 2).

Table 2. provides a detailed analysis and comparison between DCF (*n* = 21, 56.75%), PST (*n* = 8, 21.62%) and crossed pins/other screw constructs (*n* = 8, 21.62%). These groups were similar in sex, age, ethnicity, and weight, but right laterality was more common among the latter group. The duration from presentation to surgery and the surgery duration did not significantly differ between the groups, although DCF surgical time tended to be longer (*p* > 0.05, Table 2). All surgeons performed an initial closed reduction attempt, which was the ultimate reduction in 20 (54.1%) of the cases. The remaining 17 (45.9%) fractures could not reach satisfactory alignment by closed means and required an open reduction. DCF and PST were executed using similar numbers and sizes of screws and washers and similar fasciotomy rates (Table 2). Regardless of technique, patients were instructed to adhere to similar post-operative protocols, including immobilization duration, weight-bearing restrictions, and physical therapy sessions (Table 2). The immobilization was obtained by either a long leg cast or a knee immobilizer, which was defined by the surgeon’s preference.

All cases underwent union, as demonstrated on their last XR image, and the radiographic outcomes were comparable for all techniques (Table 3). The change in the coronal and sagittal alignment measurements when immediate post-operative fixation and final XR images were compared proved to be minimal (much lower than one standard deviation) and did not significantly differ between the various techniques (Table 3, *p* > 0.05 for all). The wound complications rate was minimal (*n =* 2, 5.4%), and no compartment syndrome was documented. Growth arrest rate analysis was beyond the scope of the current study due to the limited follow-up period.

## 4. Discussion

In this manuscript, the authors reviewed the surgical treatment of a SHII TTAF. To the best of their knowledge, this study represents the most extensive series published of this particular fracture subtype. This study presents DCF, a novel surgical modification in the treatment of these fractures that is more consistent with the association of osteosynthesis (AO) principles of solid fixation across both sides of a fracture. The authors found that DCF provided comparable outcomes in the short term when evaluated against PST.

Although a “bi-cortical screw” is a screw that captures two cortices, the definition of a “bi-cortical fracture fixation” should better be kept for constructs that purchase the two cortices of both fragments and should not include screws that capture two cortices of the same fragment. Having this in mind, from a fracture fixation perspective, PST, which only captures the proximal fragment cortices (and none of the distal fragment cortices), cannot by-definition provide a “bi-cortical fracture fixation” even when the screws traverse through two cortices unless these two cortices are detached. Accordingly, by this definition, a PST provides a “bi-cortical screw” but not a “bi-cortical fracture fixation” (Figure 3). DCF, corresponding to this concept, provides a bi-cortical fracture fixation, which, in theory, would provide a stronger construct.

The authors found that the short-term radiographic outcomes of the surgical treatment of SH II TTAF were excellent. Only a minimal change in alignment could be measured during the early postoperative period (from fixation to union) regardless of the method of fixation. The surgeries included open (45.9%) or closed (54.1%) reductions and internal fixation with either screws or K wires. The postoperative protocols were found to be consistent and included 4 to 6 weeks of immobilization with either a cast or a knee immobilizer in full extension and a similar period of non-weight bearing restrictions, followed by physical therapy and a gradual return to full activities (Table 1 and Table 2). The surgical treatment and postoperative protocol enabled fracture reduction to a satisfactory position that was stable enough to heal with no significant change in alignment. The patient’s features and injury mechanism in the current study were similar to comparable patient cohorts in previous reports, and the results were consistent with the existing literature [3,7,13]. Notably, the current study included 21 (56.75%) patients who have been operated on utilizing DCF, which, to our knowledge, has not been previously presented.

PST was used in 8 (21.62%) cases in the current study and has been the most published technique for fixating SH II TTAF [2,8,9,10,11,12,13] (Table 4). These publications presented excellent results and advocated performing PST. The findings in the current study align with this conclusion. Nevertheless, PST was often described as a bi-cortical fixation, even in cases where both the anterior and posterior cortices of the same fragment were captured. This definition does not accord with the suggested clarification of a “Bi-cortical fixation” and upholds a theoretical disadvantage when compared with proper bi-cortical fixation techniques such as DCF.

DCF was a very common technique in the current study (*n* = 21, 56.75%, Figure 2) and met our definition of a “bi-cortical fixation”. In Pace et al. [10], a post-operative image was presented, where a DCF was part of a multiple-direction screw construct. Nevertheless, it was not specified whether they have ever used DCF alone in any of their cases. Notably, anterior-to-posterior directed cannulated screws, such as in PST and DCF, harbor the risk of protruding the posterior cortex with the guide pins, which puts the adjacent neurovascular structures that are situated just posterior and slightly lateral to the tibia midline at risk [17]. Mun et al. found that in TTAF surgery, the more distal the screws are, the less likely are the guide pins to injure the popliteal artery if they plunge through the posterior cortex [17]. Although a rare complication, this is a potential advantage of DCF as the screws capture the posterior cortex more distally. In the current study, the outcomes of DCF were similar to the outcomes obtained with other fixation techniques (Table 3) and were comparable with previously presented outcomes in similar studies [2,8,9,10,11,12,13].

Crossed pins/screws (Figure 5) or multiple screws in multiple directions (Figure 6) were used in a minority of cases (*n* = 8, 21.62%). Crossed pins/screws allowed the purchase of four cortices on both the proximal and distal fragments. Multiple-direction screws provided stability on various planes and purchased both the distal and proximal fragment cortices. When treating profoundly unstable fractures, this screw construct remains optimal. Additionally, oblique or laterally directed fixation constructs, such as the crossed pins/screws, can minimize the risk of injuring the posterior neurovascular structures [17]. Of note, in both methods, the implants were positioned in very close proximity to the tibial articular surface. Therefore, care should be taken not to compromise the articular surface.

A total of 75.7% of the current study participants were documented to have obtained a full range of motion after surgery within a short follow-up period, and this rate would have been expected to grow significantly with a longer follow-up period. The complication rate for SH II TTAF has been previously reported to be 20% [3]. Both Park et al. [9] and Formicini et al. [12] reported on a full functional recovery of their patients with a long-term follow-up. Pace et al. [10] reported that all of their 24 cases underwent a clinical and radiographic union, although three patients developed a massive distal vein thrombosis )DVT(, a CS, and a meniscal tear. Brey et al. [7] pointed out that fractures with a posterior Thurston–Holland component were a sub-group that was more susceptible to harboring complications. Since they combined both mWJ 4 and 5 in one group, comparison with the current study was limited. The follow-up duration in the current study was not sufficient to draw robust conclusions on the total complication rates in the surgical treatment of these fractures.

Periosteal entrapment might interfere with SH II TTAF reduction [9]. In the current study, 20 (54.1%) cases could be successfully reduced without exposing the fracture site. Park et al. [9] pointed out that a periosteal entrapment was observed in the anteromedial aspect of proximal tibial physis on MRI in all 10 patients they reviewed. They were able to obtain a satisfactory reduction only once the entrapped periosteum was repositioned. We agree with Park et al. that surgeons should strive for an anatomic reduction, and while in the current study, many fractures could be successfully reduced by closed means, when deemed necessary, an open surgical approach that allows addressing obstacles to reductions is mandatory.

Compartment syndrome (CS) has been previously associated with SH II TTAF [13]. In the current study, a prophylactic fasciotomy was not routine but was performed in 5 (13.5%) cases, and CS was never diagnosed postoperatively. Nevertheless, it was common (*n* = 8, 21.6%) to find a statement in the operative note describing that the injury had violated the anterior fascia, which was found to be torn, and, therefore, no additional fasciotomy was performed. Haber et al. stated that about a quarter of their 236 mWJ 1 to 5 cases had been treated with a prophylactic fasciotomy. CS was identified in 3 (11%) of their 26 SH II TTAF cases. Pace et al. [10] reported that 1 of their 24 patients was pre-operatively diagnosed with CS. They suggested that a routine fasciotomy might be required in SH II TTAF. Park et al. [9] did not include fasciotomy in their surgical description, but they did state that none of their 10 patients had been diagnosed with CS. Although we were not able to find clear evidence for the necessity of performing a prophylactic fasciotomy in the current study, previous literature suggests that addressing this possibly devastating complication is of utmost importance. The authors of this study are in complete agreement with this statement.

This study had limitations. It had the inherent weaknesses of a retrospective study. The surgical techniques were not standardized, nor were they randomly allocated. The fluoroscopy and follow-up XR images were not standardized either. Our case identification strategy focused on identifying surgically treated patients only. Accordingly, it was beyond the scope of this article to report the rate of surgical vs. non-surgical treatment of SH II TTAF. Given that our primary focus was to elucidate the technical aspects of fracture fixation, our analysis emphasized the surgery-to-fusion period, prompting a relatively brief follow-up period. This limited our ability to investigate the incidence and pattern of growth arrest as a possible complication, and the rate of hardware irritation and removal, which would normally take place at a later postoperative point in time. Clinical outcomes were extracted from medical records documentation, and systematic patient-reported outcome measures were not available. The routine postoperative protocol was very protective. Therefore, the fracture fixation constructs were not significantly challenged. This limited our ability to evaluate the fixation stability, and the superiority of either fixation method could not be proven. Nevertheless, the safety and efficacy of the DCF could be appreciated. A biomechanical study that evaluates the fixation stability of the various fixation techniques is needed to address this limitation.

## 5. Conclusions

The DCF, which has theoretical biomechanical advantages, was presented and discussed for the first time in literature. The surgical treatment of Salter–Harris II tibial tubercle avulsion fractures, including DCF, was presented and was found to provide satisfactory union rates on a short-term follow-up. 

## Figures and Tables

**Table 1:** Tibial tuberosity avulsion fractures type 4 modified Watson–Jones treated operatively. DCF: Distal Cortical Fixation; PST: Proximal screw technique; Crossed/other: Crossed pins or screws or screws inserted in multiple directions.

Materials and Methods									Results		
	Technique Group	Sex	Age	Side	Injury Setting/Mechanism	Follow-Up Duration	NWB (Weeks)	Immobilization Duration (Weeks)	Wound Complications	Change in Coronal Alignment (Degrees)	Change in Sagittal Alignment (Degrees)
1	DCF	F	13.98	L	Running in soccer	89	5	5	None	−0.60	1.80
2	DCF	F	13.87	R	fall	24	3	3	None	2.60	0.90
3	DCF	F	13.84	L	Jumping in gymnastics	74	6	6	None	−0.50	1.40
4	DCF	M	17.23	L	Foot caught	449	6	6	None	−1.00	0.80
5	DCF	M	14.01	L	Trampoline	33	4	6	None	0.00	0.30
6	DCF	M	15.20	L	Basketball, hit knee on a pole	605	7	7	None	2.00	0.70
7	DCF	M	14.66	L	Jumping in basketball	140	5	5	None	0.50	−0.80
8	DCF	M	15.51	R	Football, while Running	89	5	5	None	3.10	1.40
9	DCF	M	15.83	L	Kicked in leg playing soccer	209	4	4	None	−2.10	−3.00
10	DCF	M	12.44	L	Ran and felt a “pop”	60	4	4	None	0.80	−5.10
11	DCF	M	15.59	L	Jumping in basketball	15	2	no sufficient follow-up	None	0.80	−0.70
12	DCF	M	18.16	L	Playing basketball	36	5	5	None	1.00	−1.70
13	DCF	M	14.37	r	Jumping at basketball	775	2	4	Serous drainage 2 years after surgery	−3.00	−3.10
14	DCF	M	13.72	L	Running in soccer	179	0	3	None	−3.40	−4.30
15	DCF	M	15.22	R	Felt a “pop” while running	583	6	4	None	0.50	1.50
16	DCF	M	15.23	L	Running in basketball	78	4	4	None	−0.10	0.20
17	DCF	M	15.66	R	Jumping in basketball	155	0	4	None	−1.60	−0.50
18	DCF	M	14.04	L	Playing bsketball	289	5	5	None	1.20	3.20
19	DCF	M	14.68	L	Abrupt stop while running	31	6	4	None	4.10	−2.20
20	DCF	M	16.24	L	Jumping in basketball	391	7	7	None	1.50	−1.50
21	DCF	M	12.97	L	Running in dodgeball, head a “pop”	836	4	3	None	3.00	−9.30
22	PST	M	14.12	R	Jumping at basketball	157	4	4	None	−4.30	−3.30
23	PST	M	14.64	L	Jumping rope	194	4	4	None	1.60	0.10
24	PST	M	13.91	L	Trampoline park	240	4	4	None	1.50	−10.40
25	PST	M	12.72	Bil	Slipped on water playing basketball	366	6	6	wound dehiscence	1.60	−2.40
26	PST	M	15.68	L	Jumping hurdles	117	6	6	None	1.70	3.10
27	PST	M	12.87	R	Trampoline	41	7	3	None	−1.00	unmeasurable
28	PST	M	15.59	L		72	7	7	None	−0.10	1.10
29	PST	M	14.76	R	Running/“roughhousing”	468	2	8	None	2.70	1.40
30	Crossed	M	15.50	R	Jumping in basketball	97	4	4	None	2.60	2.80
31	Crossed lateral and medial	M	14.09	L	Soccer	98	2	7	None	1.70	−4.10
32	Crossed lateral and medial	M	16.35	R	Jumping on trampoline	141	4	4	None	−1.30	8.60
33	Crossed lateral and medial	M	15.85	R	Stepped wrong on ladder, fell, and hit his knee	26	6	3.5	None	2.60	3.10
34	Crossed lateral and medial	M	15.50	R	Jumping in basketball	103	4	2	None	0.10	2.20
35	Multiple	M	15.30	R	Jumping over fence	366	0	6	None	−1.50	0.20
36	Multiple	M	15.84	R	Jumping in basketball	358	6	6	None	−1.60	1.00
37	Multiple	M	13.79	L	Fell while running in baseball	343	4	4	None	−0.20	−2.60

**Table 2:** A univariate analysis comparing surgical techniques for tibial tuberosity avulsion fractures type 4 modified Watson–Jones treated operatively. DCF: Distal Cortical Fixation; PST: Proximal Screw Technique; Crossed/other: Crossed pins or screws or screws inserted in multiple directions. * Although not all fluoroscopy studies proved a bi-cortical fixation, most operative reports stated that the posterior cortex was purchased. This discrepancy might originate from the round shape of the tibia and screws that were not directed in an exact anterior-to-posterior direction. The researchers used the fluoroscopy images for the current table formation and analysis.

Variable	DCF (*n* = 21, 56.75%)	PST (*n* = 8, 21.62%)	Crossed/Other (*n* = 8, 21.62%)	Total (*n* = 37, 100%)	*p* Value
Materials and Methods					
**Sex (Male)**	18 (85.7%)	8 (100%)	8 (100%)	34 (91.9%)	0.395
**Age (Years)**	14.88 ± 1.35	14.28 ± 1.11	15.28 ± 0.88	14.83 ± 1.23	0.263
**Side (Left)**	16 (76.2%)	5 (62.5%)	2 (25%)	23 (62.2%)	0.059
**Ethnicity**					0.644
**Black**	9 (42.9%)	4 (50%)	5 (62.5%)	18 (48.6%)	
**White**	9 (42.9%)	2 (25%)	3 (37.5%)	14 (37.8%)	
**Hispanic/Asian/other**	3 (14.3%)	2 (25%)	0 (0%)	5 (13.5%)	
**Weight (kg)**	78.73 ± 20.35	75.42 ± 17.63	74.43 ± 19.42	77.09 ± 19.17	0.888
**Weight percentile**	84.34 ± 19.96	95.76 ± 4.79	76.26 ± 24.9	84.77 ± 19.91	0.14
**Time from presentation to surgery**	1.57 ± 2.29	2.75 ± 3.41	1.13 ± 0.354	1.73 ± 2.29	0.679
**Number of patients who underwent additional imaging studies**					0.004
**CT**	1 (4.8%)	3 (37.5%)	4 (50%)	8 (21.6%)	
**MRI**	0 (0%)	1 (12.5%)	0 (0%)	1 (2.7%)	
**Duration of surgery (minutes)**	80.05 ± 26.13	68.88 ± 28.59	57.25 ± 11.89	72.5 ± 25.59	0.102
**Closed reduction (vs. open reduction)**	10 (47.6%)	4 (50%)	6 (75%)	20 (54.1%)	0.45
Number of screws per surgery *					0.014
**1**	2 (9.5%)	1 (12.5%)	0 (0%)	3 (8.1%)	
**2**	14 (66.7%)	5 (62.5%)	1 (12.5%)	20 (54.1%)	
**3**	5 (23.8%)	2 (25%)	3 (37.5%)	10 (27%)	
**4**	0 (0%)	0 (0%)	1 (12.5%)	1 (2.7%)	
**K wires**	0 (0%)	0 (0%)	3 (37.5%)	3 (8.1%)	
**Screw size**					0.006
**4.0**	3 (14.3%)	0 (0%)	0 (0%)		
**4.5**	15 (71.4%)	5 (62.5%)	1 (12.5%)		
**6.5**	2 (9.5%)	2 (25%)	2 (25%)		
**Mixed**	1 (4.8%)	1 (12.5%)	2 (25%)		
**K wires**	0 (0%)	0 (0%)	3 (37.5%)		
**Washer (yes)**	12 (57.1%)	4 (50%)	1 (12.5%)	17 (45.9%)	0.115
**Biocortical purchase**	13 (61.9%) *	8 (100%)	8 (100%)	29 (78.4%)	0.021
**Fasciotomy**					0.814
**Surgical**	2 (9.5%)	2 (25%)	0 (0%)	4 (10.8%)	
**Traumatic (documented)**	5 (23.8%)	1 (12.5%)	1 (12.5%)	7 (18.9%)	
**Both**	1 (4.8%)	0 (0%)	0 (0%)	1 (2.7%)	
**Follow-up duration (weeks)**	35.17 ± 36.79	30.03 ± 20.84	28 ± 19.58	32.51 ± 30.34	0.883
**Duration of complete immobilization at full extension (weeks)**	4.7 ± 1.22	5.25 ± 1.75	4.56 ± 1.63	4.79 ± 1.42	0.707
**Duration of non-weight-bearing restriction (weeks)**	4.29 ± 1.98	5 ± 1.77	3.75 ± 1.98	4.32 ± 1.93	0.435
**Referral to physical therapy**	21 (100%)	8 (100%)	7 (87.5%)	36 (97.3%)	0.432
**Results**					
**Documented pain after 3 months**					0.517
**Yes**	4 (19%)	2 (25%)	0 (0%)	6 (16.2%)	
**No**	13 (61.9%)	6 (75%)	7 (87.5%)	26 (70.3%)	
**Lacking sufficient follow-up**	4 (19%)	0 (0%)	1 (12.5%)	5 (13.5%)	
**Wound complications**	0 (0%)	1 (4.8%)	1 (12.5%)	2 (5.4%)	0.685
**Full range of motion on last follow-up**					0.761
**Yes**	15 (71.4%)	7 (87.5%)	6 (75%)	28 (75.7%)	
**No**	3 (14.3%)	0 (0%)	0 (0%)	3 (8.1%)	
**Lacking sufficient follow-up**	3 (14.3%)	1 (12.5)	2 (25%)	6 (16.2%)	

**Table 3:** The coronal and sagittal plane alignment of tibial tuberosity avulsion fractures type 4 modified Watson–Jones following a surgical intervention. DCF: Distal Cortical Fixation; PST: Proximal screw technique; Crossed/other: Crossed pins or screws or screws.

Variable	DCF (*n* = 21, 56.75%)	PST (*n* = 8, 21.62%)	Crossed/Other (*n* = 8, 21.62%)	Total (*n* = 37, 100%)	*p* Value
**Coronal alignment (degrees)**					
**Immediate postoperative**	88.2 ± 1.86	86.96 ± 2.08	86.47 ± 1.41	87.56 ± 1.93	
**Last documented (fused fracture)**	87.78 ± 1.57	86.5 ± 1.48	86.17 ± 2.6	87.16 ± 1.91	
**Difference**	0.41 ± 1.97	0.46 ± 2.24	0.3 ± 1.78	0.4 ± 1.94	0.872
**Sagittal alignment (degrees)**					
**Immediate postoperative**	9.7 ± 4.95	9.91 ± 7.84	8.85 ± 4.68	9.55 ± 4.94	
**Last documented (fused fracture)**	8.75 ± 3.58	8.11 ± 5.01	10.25 ± 4.18	8.93 ± 3.99	
**Difference**	0.95 ± 2.86	1.48 ± 4.52	−1.4 ± 3.87	0.53 ± 3.51	0.296

**Table 4:** A representation of a Proximal Screw Technique (PST) for fixating type 4 modified Watson–Jones fractures in the literature.

Refs.	mWJ4 Cases (n)	Relevant Findings and Conclusions
**Pace et al.** [9]	24	The authors’ perception was that longer bi-cortical screws were necessary to stabilize the posterior fracture fragment. Accordingly, they stated that a screw purchase in this fragment should be achieved whenever possible. In their series, 4 patients required a supplemental plate fixation.
**Haber et al.** [12]	26	In all, 79% of the mWJ4/5 fractures were treated operatively. The authors did not separate types 4 and 5 in their analysis. Although they did not elaborate on the technique in the text, the image provided by the authors presented a PST as their surgical method.
**Arkader et al.** [10]	13	A total of 12 cases were operated with uni-cortical fixation, while only 1 case was treated with a mixed uni- and bi-cortical fixation. The authors concluded that a uni-cortical fixation might be suitable in mWJ4 fractures.
**Park et al.** [14]	10	All fractures were fixated by a PST construct utilizing 2 (in a few cases) or 3 (in most cases) screws, most commonly 6.5 mm cancellous. The entry points were medial and lateral to the tibial tuberosity without violating the tibial apophysis.
**Formicini et al.** [11]	5	The authors used 4.5 mm cannulated screws and pointed out that while mWJ 1–3 could be treated with uni-cortical screws, mWJ4 fractures required greater stability, especially for the posterior component. For this reason, they used bi-cortical screws that engaged this component to form a construct regarded by them to be more effective.
**Rodriguez et al.** [7]	review	The authors presented a review of tibial tuberosity fractures. In their review, they did not discuss the specifications of the screw trajectory but did provide an image that represented their concept of a proper screw position for fixating mWJ4 fractures. In that image, the posterior component was captured with a PST, with two fully threaded screws that were inserted through the tibial tuberosity midline.

## Data Availability

The data that support the findings of this study are not openly available due to reasons of sensitivity and privacy and are available from the corresponding author upon reasonable request.
